# Variable radiographic and histologic presentations of amiodarone‐related interstitial lung disease and the importance of avoiding re‐exposure

**DOI:** 10.1002/rcr2.1165

**Published:** 2023-05-25

**Authors:** I‐Lin Tsai, Li‐Ting Huang, Yu‐Ting Yu, Chung‐Ta Lee, Tang‐Hsiu Huang

**Affiliations:** ^1^ Division of Chest Medicine, Department of Internal Medicine National Cheng Kung University Hospital, College of Medicine, National Cheng Kung University Tainan Taiwan; ^2^ Department of Diagnostic Radiology National Cheng Kung University Hospital, College of Medicine, National Cheng Kung University Tainan Taiwan; ^3^ Department of Pathology National Cheng Kung University Hospital, College of Medicine, National Cheng Kung University Tainan Taiwan

**Keywords:** amiodarone, interstitial lung diseases, pneumonia

## Abstract

Amiodarone is a commonly used antiarrhythmic agent but exhibits potential pulmonary toxicity. In this case series, we describe the clinical, radiographic, and histologic manifestations of three patients who developed interstitial lung disease (ILD) following amiodarone treatment for variable lengths of time with different dosages. The presentations on computed tomographic images and in pulmonary pathology differed among the three patients. All three had immediate discontinuation of amiodarone and received treatment with systemic corticosteroids. One patient eventually died from ventilator‐associated pneumonia after an initial improvement. The other two patients recovered well but later experienced ILD recurrence following brief re‐exposure to amiodarone. Through this case series, we aim to demonstrate the variable features of amiodarone‐related ILD, and highlight the importance of timely amiodarone cessation and avoiding re‐exposure to prevent the progression and recurrence of ILD.

## Graphical abstract

Amiodarone‐related interstitial lung disease may exhibit variable clinical, radiographic, and histologic presentations. Prompt discontinuation and avoiding re‐exposure of the agent is important to prevent the progression and recurrence of the interstitial lung disease.

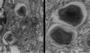

## INTRODUCTION

Amiodarone is a commonly used antiarrhythmic agent, owing to its efficacy and the convenience in its administration and dosing titration. However, the agent exhibits potential toxicity to multiple organs including the lungs, and there have been reports of respiratory complications following treatment with amiodarone even at low doses.^1^, ^2^, ^3^ In this case series, we describe the manifestations of three patients with histology‐proven amiodarone‐related interstitial lung diseases (ILD). We aim to demonstrate the variable radiographic and histologic features of amiodarone‐related ILD and highlight the importance of avoiding further amiodarone exposure to these patients.

## CASE SERIES

### Case 1

**FIGURE 1:**
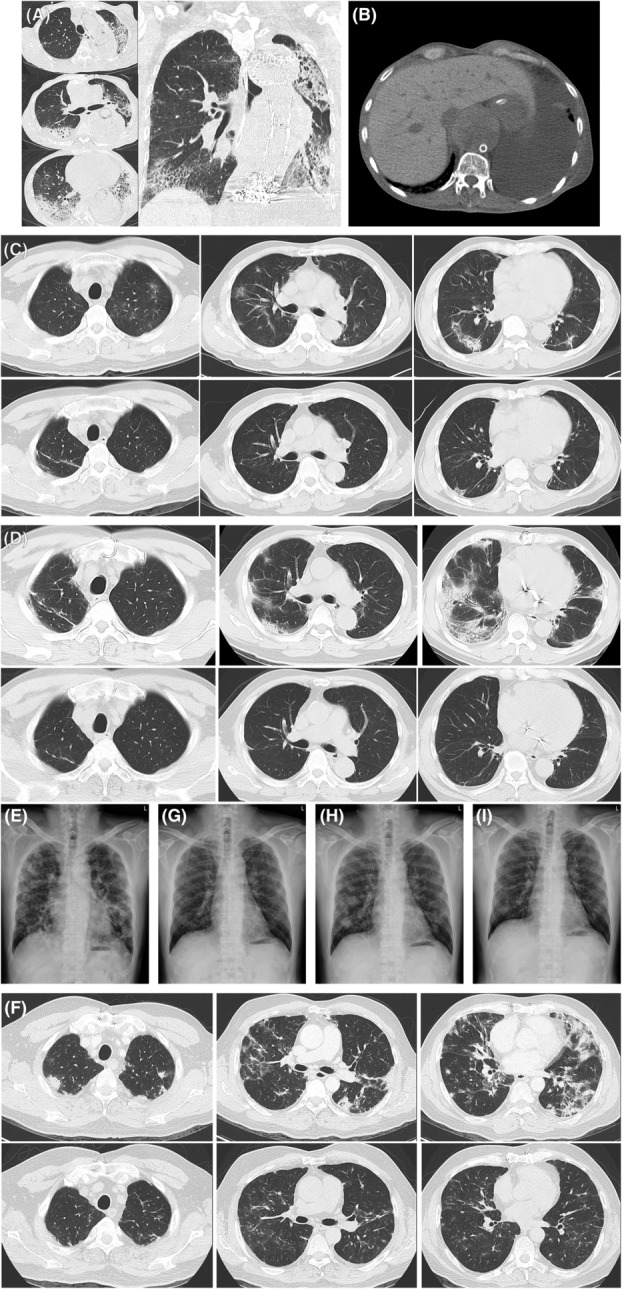
(A) Computed tomographic (CT) images of case 1 in transverse and coronal views. (B) Diffuse hyperintensity of the liver on the initial CT image of case 1. (C) Initial (upper 3 panels) and follow‐up (lower 3 panels) CT images of the first episode of interstitial lung disease (ILD) of case 2. (D) Initial (upper 3 panels) and follow‐up (lower 3 panels) CT images of the second (recurrent) episode of ILD of case 2. (E) The initial chest radiograph of case 3. (F) Initial (upper 3 panels) and follow‐up (lower 3 panels) CT images of the first episode of ILD of case 3. Serial subsequent chest radiographs of case 3, showing: (G) post‐treatment resolution of the first episode of ILD; (H) recurrent ILD following amiodarone re‐exposure; (I) resolution of the recurrent ILD.

**FIGURE 2:**
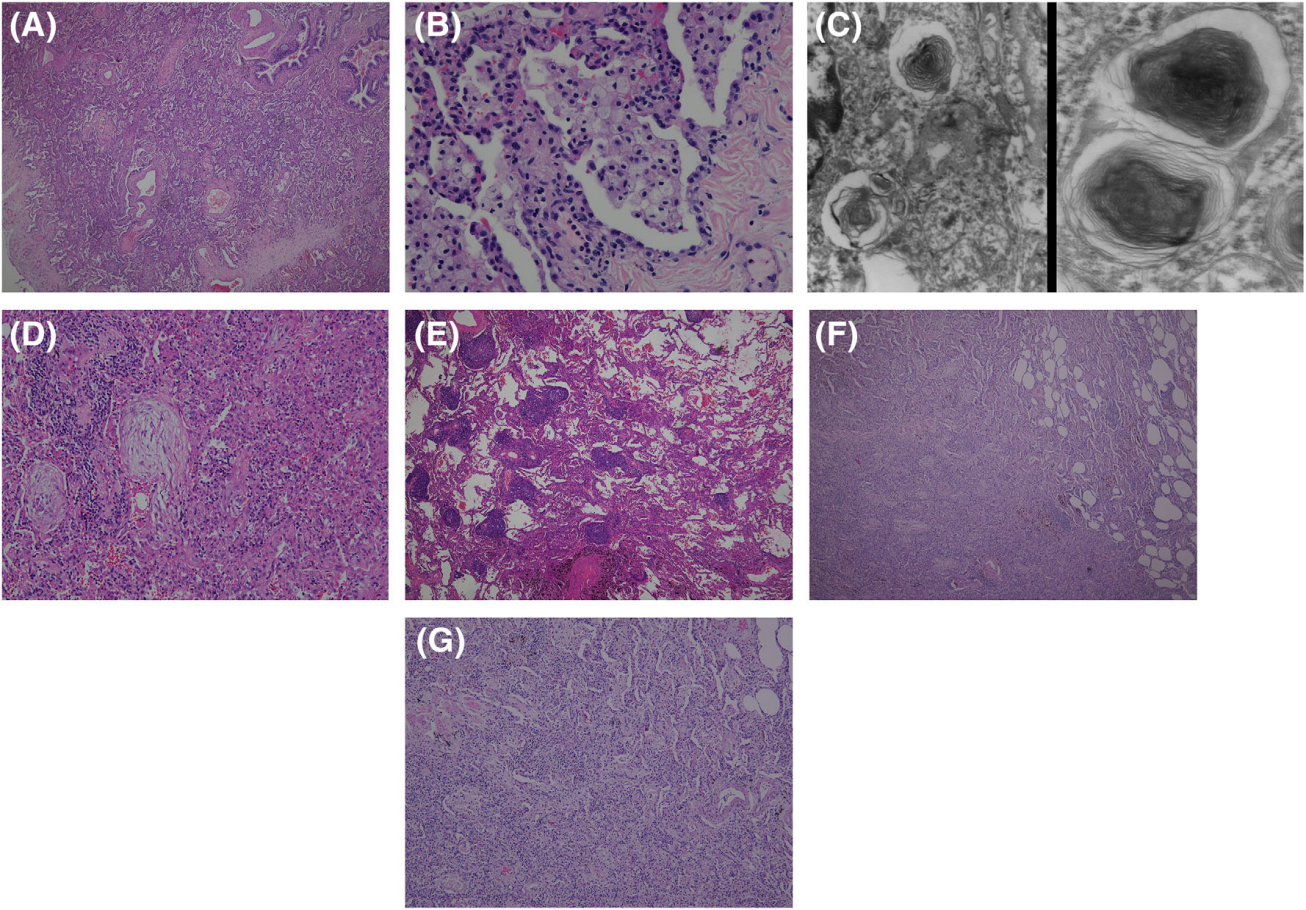
Histologically case 1 exhibited (A) a prominent pattern of nonspecific interstitial pneumonia (NSIP; H & E stain; 40×) and (B) many intra‐alveolar clusters of foamy macrophages (H & E stain; 1000×) containing (C) cytoplasmic lamellar inclusion bodies (electron microscopy images; left panel 30,000×; right panel 60,000×). Case 2 exhibited (D) features of organizing pneumonia with intra‐alveolar Masson bodies (H & E stain; 100×) and (E) features of lymphocytic interstitial pneumonia with dense lymphocytic infiltration and lymphoid aggregates (H & E stain; 40×). Case 3 exhibited (F) mixed patterns containing features of organizing pneumonia and NSIP (H & E stain; 40×), wherein (G) intra‐alveolar Masson bodies and clusters of foamy macrophages can be discerned (H & E stain; 100×).

A 62‐year‐old man was transferred to our hospital in August 2020, presenting with rapidly progressive bilateral pneumonia and the associated acute respiratory failure. The initial thoracic computed tomography (CT) showed ground‐glass opacities and dense consolidations involving both lungs (Figure 1A) and diffuse hyperintensity of the liver (Figure 1B). There was no clinical or serological evidence of coexisting connective tissue diseases (CTDs). All the microbiological surveys came back with negative results. Pneumonia responded poorly to broad‐spectrum antibiotics. Further inquiry of his past histories revealed that the patient had been taking amiodarone (400 mg/day; prescribed at another hospital) for atrial fibrillation (Af) in recent 6 months until this admission. Wedge‐biopsy histology (from the left upper lobe) reported features of nonspecific interstitial pneumonia (NSIP) (Figure 2A) together with many intra‐alveolar clusters of foamy macrophages (Figure 2B). Lamellar inclusion bodies were observed in these macrophages under electron microscopy (Figure 2C). Although the patient experienced a transient improvement in his respiratory conditions following the treatment with high‐dose intravenous methylprednisolone (40 mg/day for a body weight of 43 Kg), he later died from ventilator‐associated pneumonia.

### Case 2

A 72‐year‐old non‐smoking male had his first episode of amiodarone‐related ILD in May 2012, after 18 months of amiodarone therapy (600 mg/day for 30 days, then 200 mg/day) for Af. His symptoms were relatively mild (including cough and exertional dyspnea). Serological surveys reported negative results for CTD‐related autoantibodies. The thoracic CT revealed multiple scattering ground‐glass and alveolar‐consolidating opacities and ill‐defined nodules in bilateral lungs (Figure 1C upper 3 panels). Wedge‐biopsy histology (from his right upper and lower lobes) reported combined features of organizing pneumonia and lymphocytic interstitial pneumonia (Figure 2D, E). Clustering of foamy macrophages, however, was not observed. His ILD gradually resolved after shifting to dronedarone and a course of low‐dose (10–15 mg/day for a body weight of 59 Kg with gradual tapering) oral prednisolone (Figure 1C lower 3 panels). The patient later received a repairment surgery on his mitral valve in 2013. In June 2020, less than 2 months after the cardiologist changed back to amiodarone (200 mg/day), he experienced a symptomatic recurrence of the ILD, which appeared even more extensive radiographically than in the previous episode (Figure 1D upper 3 panels). No specific microbial pathogen was isolated. The recurrence resolved over 4 months after promptly shifting amiodarone to rytmonorm and another course of low‐dose (15 mg/day for a body weight of 60 Kg with gradual tapering) oral prednisolone (Figure 1D lower 3 panels).

### Case 3

A 65‐year‐old non‐smoking male had been treated with amiodarone (100–200 mg/day) for paroxysmal Af since February 2019. In May 2020, he presented with a non‐productive cough, exertional dyspnea, and hypoxemia (with the pulse oximetry measuring 89%–92%) while breathing ambient air that had progressively aggravated over several weeks. He was afebrile and exhibited no symptoms, signs, or serological evidence of CTDs. The chest radiograph showed extensive ill‐defined opacities in both lung fields (Figure 1E), while the CT scan revealed multiple alveolar‐consolidating and ground‐glass opacities, ill‐defined nodules, and thickened septal lines in his both lungs (Figure 1F upper 3 panels). All the microbiological surveys (including those for SARS‐CoV‐2) reported negative findings. Wedge‐biopsy histology (from his left upper lobe) reported features of organizing pneumonia combined with NSIP and clusters of foamy macrophages in the alveolar space (Figure 2F, G). The patient recovered well after stopping amiodarone and a course of moderate‐dose oral prednisolone (40 mg/day for a body weight of 68 Kg) that was tapered off over 6 months (Figure 1F lower 3 panels and 1G). However, amiodarone treatment was resumed by the cardiologist in December 2022 because the patient still had intermittent palpitation (despite electrophysiological ablations and a rate‐control therapy with diltiazem). Within weeks, he once again developed a non‐productive cough; multiple new opacities appeared on the follow‐up chest radiograph (Figure 1H). Amiodarone was immediately replaced by flecainide and verapamil. Without the use of corticosteroid, the patient's cough quickly improved, and a chest radiograph 4 weeks later showed significant resolution of the abnormal opacities (Figure 1I).

## DISCUSSION

Amiodarone exhibits potential pulmonary toxicity, though the exact pathogenesis is still not fully elucidated. Proposed mechanisms of alveolar epithelial injury (which elicits subsequent inflammatory reactions) include intracellular oxidative stress relating to the iodine‐moiety of amiodarone, impaired organelle functions and phospholipid metabolism due to the amphophilic structure of amiodarone, dysregulated autophagy, and epithelial apoptosis possibly mediated by angiotensin‐2 associated pathways.^1^, ^4^, ^5^, ^6^, ^7^ Reported risk factors for developing amiodarone‐related pulmonary toxicity are advanced age (older than 60 years), coexisting heart failure, prolonged treatment (>6 months), high daily or accumulated dosage, and recent cardiac surgery or exposure to iodine‐containing contrast medium.^1^, ^2^, ^3^, ^8^ In retrospect, all three patients in this present case series were more than 60 years old and received amiodarone for more than 6 months when the ILD occurred. Nevertheless, the daily dosage (particularly that of case 3) was not necessarily high, which implies that clinicians must remain vigilant to the occurrence of ILD during amiodarone therapy regardless of the daily maintenance dosing regimen.^2^, ^3^

The CT features of all three patients were compatible with that of organizing pneumonia with mixed patterns and multi‐lobe involvement. It has been reported recently that organizing pneumonia that manifests on CT images as mixed patterns and extensive involvement is more likely to be secondary than cryptogenic when compared to organizing pneumonia presenting solely with limited alveolar consolidation.^9^ Such CT features should prompt further investigations to identify the causative aetiology (which was amiodarone‐related pulmonary toxicity for our three patients). The histologic features were relatively heterogeneous among the three cases. Clusters of foamy macrophages were frequently seen in cases 1 and 3 but not in case 2. Organizing pneumonia was the main histologic pattern in cases 2 and 3. Nevertheless, coexisting features of lymphocytic interstitial pneumonia, and features of NSIP, were also present in case 2 and case 3, respectively. In contrast to the CT images displaying mainly alveolar‐filling opacities without subpleural sparing, histology of the biopsied lung tissue in case 1 showed a predominant pattern of NSIP. This discrepancy between CT images and histology might have resulted from the site selection for biopsy, as organizing pneumonia and NSIP may coexist.^10^, ^11^ The heterogeneity in the pulmonary histology of our patients supports the rationale that a single drug can potentially cause different pulmonary histologic reactions simultaneously in the same individual. It has been suggested that, when approaching a newly diagnosed ILD that presents with mixed histological patterns, it is important to exclude the possibility of drug‐induced or CTD‐related ILD.^11^

Case 1 presented a rapid and severe form of ILD, whereas the ILDs in cases 2 and 3 exhibited a relatively subacute course. These differences demonstrate the wide variability in the severity and presentations of drug‐induced ILDs among different individuals. Moreover, case 2 and case 3 had a recurrence of the ILD after re‐exposure to amiodarone. Even though case 2 had already stopped taking amiodarone for more than 8 years, the re‐exposure quickly led to a recurrent ILD that appeared even more extensive than in the first episode. Furthermore, the recurrent ILD in case 3 quickly resolved after the mere discontinuation of amiodarone, even without corticosteroid treatment. These clinical features of our patients highlight the key point in managing drug‐induced ILD, which is the prompt removal of the triggering agent and the avoidance (as possible) of any subsequent re‐exposure.

In conclusion, we describe in this case series the different clinical, CT, and histologic presentations of three cases of amiodarone‐related ILDs, which can be quite variable from patient to patient. Clinicians treating patients with amiodarone must be alert to the potential pulmonary toxicity of this agent regardless of the dosing regimen and involve amiodarone‐related ILD as a major differential diagnosis when the patients exhibit unexplained respiratory or radiographic abnormalities. For patients who develop ILD, timely discontinuation of amiodarone and the avoidance of future re‐exposure is critical.

## AUTHOR CONTRIBUTIONS

I‐Lin Tsai and Tang‐Hsiu Huang contributed to the concept, clinical data collection and review, and the drafting of the manuscript. Li‐Ting Huang contributed to the collection and curation of radiographic images and the editing of the pertinent descriptions. Yu‐Ting Yu and Chung‐Ta Lee contributed to the collection and curation of histological and electron microscopy images and the editing of pertinent descriptions. Tang‐Hsiu Huang contributed to the critical review of the manuscript and the correspondence.

## CONFLICT OF INTEREST STATEMENT

None declared.

## ETHICS STATEMENT

The authors declare that appropriate written informed consent was obtained for the publication of this manuscript and accompanying images.

## DISCLOSURES

Parts of the information described in this case series were presented in a thematic poster in the 2022 Annual Congress of Taiwan Society of Pulmonary and Critical Care Medicine.

## Data Availability

Data available on request from the authors.
